# The Effect of 1.5 Tesla MRI on Microleakage and Thermal Stability of Stainless Steel and Titanium Orthodontic Brackets: An *in vitro* Study 

**DOI:** 10.30476/dentjods.2025.105157.2574

**Published:** 2025-12-01

**Authors:** Maryam Paknahad, Yasaman Ghaedi, Fatemeh Hajipour, Shabnam Ajami

**Affiliations:** 1 Oral and Dental Disease Research Center, Dept. of Oral and Maxillofacial Radiology, School of Dentistry, Shiraz University of Medical Sciences, Shiraz, Iran.; 2 Student Research Committee, School of Dentistry, Shiraz University of Medical Sciences, Shiraz, Iran.; 3 Postgraduate Student, Dept. of Orthodontics, School of Dentistry, Shiraz University of Medical Sciences, Shiraz, Iran.; 4 Orthodontic Research Center, School of Dentistry, Shiraz University of Medical Science, Shiraz, Iran.

**Keywords:** Orthodontics, Orthodontic Bracket, Magnetic Resonance Imaging, Adhesive, Temperature

## Abstract

**Background:**

Fixed orthodontic appliances, such as stainless steel and titanium brackets, might become exposed to magnetic resonance imaging (MRI) during treatment. However, the effects of MRI on microleakage and thermal changes in these brackets have not been thoroughly investigated. This study addresses these gaps to ensure safety and efficacy in patients undergoing orthodontic treatment while exposed to MRI.

**Purpose:**

This study investigates and compares the effects of 1.5 tesla (T) MRI exposure on microleakage and temperature changes in stainless steel and titanium orthodontic brackets, evaluating their safety and bond integrity during orthodontic treatment.

**Materials and Method:**

In this *in vitro* study, forty non-carious, freshly extracted human maxillary premolars were randomly divided into stainless steel and titanium bracket groups (n=20). Each group was further subdivided into MRI-exposed (case) and non-exposed (control) subgroups. The case subgroups were subjected to a 1.5 T MRI scan for 20 minutes. Microleakage was evaluated using dye penetration under a stereomicroscope, and temperature changes were measured before and after MRI exposure. Statistical analysis included Kruskal-Wallis tests and paired t-tests. Significance was set at *p* Value <0.05.

**Results:**

Microleakage at the enamel-adhesive interface was slightly higher than at the bracket-adhesive interface in all groups, but the differences were not statistically significant (*p*> 0.05). No significant differences in microleakage or temperature changes were observed between stainless steel and titanium brackets following MRI exposure (*p*> 0.05).

**Conclusion:**

Exposure to a 1.5 T MRI magnetic field does not significantly affect microleakage or temperature changes in stainless steel or titanium brackets. These findings suggest that fixed orthodontic appliances do not need to be removed prior to MRI examinations, provided artifacts or image interference are not a concern.

## Introduction

Magnetic resonance imaging (MRI) is a sophisticated imaging modality that uses strong magnetic fields to generate high-resolution images of biological tissues [ 1 - 3 ]. Unlike computed tomography (CT) and cone beam computed tomography (CBCT), MRI provides detailed visualization of soft tissue structures and offers the significant advantage of avoiding ionizing radiation, making it a preferred tool for non-invasive diagnosis of soft tissue diseases [ 4 - 5 ]. It is widely used in managing head and neck disorders, particularly for detailed visualization of the temporomandibular joint and other soft tissue structures . Recent advancements indicate that MRI holds significant potential for future applications in orthodontics, such as MRI-based cephalometric analysis, which could revolutionize treatment planning [ 10 - 11 ]. 

Dentoalveolar malalignments are prevalent across populations. Orthodontic treatments using fixed appliances are a common method to correct various dental and jaw abnormalities [ 12 ]. However, the increasing number of orthodontic patients undergoing MRI has raised concerns regarding the compatibility of these appliances with strong magnetic fields [ 13 - 14 ]. Metallic components, whether ferromagnetic or not, can become magnetized to varied degrees based on their magnetic susce-ptibility and may interact with MRI fields [ 15 - 16 ]. These interactions can result in several challenges, including signal loss due to metal artifacts, which can compromise image interpretation and lead to misdiagnosis [ 13 , 17 ]; mechanical effects such as translational attraction and torque, potentially displacing metallic appliances [ 18 ]; and localized tissue heating induced by electrical currents generated within the metallic objects [ 19 - 20 ]. 

Orthodontic appliances, especially brackets, are made from a variety of materials, including metallic alloys such as nickel (Ni), titanium (Ti), and stainless steel, as well as non-metallic components like ceramic and plastic [ 21 ]. However, metallic materials remain the primary choice for constructing most orthodontic brackets due to their superior physical strength and ease of shaping into diverse forms [ 22 ]. Stainless steel brackets, due to their ferromagnetic properties, are more likely to cause significant imaging artifacts and magnetic attraction compared to titanium brackets, which are largely non-ferromagnetic [ 23 - 26 ]. 

The presence of an orthodontic appliance in a patient's mouth can create a potentially unsafe situation due to interactions with the MRI magnetic field [ 15 ]. The immediate risk is the attraction between the MRI magnet and ferromagnetic metal components, which can lead to displacement or movement of the orthodontic appliance. In addition to causing imaging artifacts, metallic objects may also experience heating effects due to the electromagnetic field generated by the MRI [ 20 ]. 

One important aspect of the interaction between orthodontic treatment and MRI is the potential displacement of metallic orthodontic components, which may occur due to translational attraction or torque forces induced by the magnetic field [ 27 ]. This phenomenon is particularly critical in the case of brackets; as such displacements can weaken the bond strength and increase the risk of microleakage or debonding. The presence of ferromagnetic elements in orthodontic brackets may contribute to these effects by inducing localized mechanical stress at the bracket–enamel interface, ultimately compromising the integrity of the adhesive bond [ 25 ]. Microleakage is clinically significant as it can lead to white spot lesions, enamel decalcification, and potential bracket debonding during treatment [ 28 ]. Despite these potential risks, it is not always feasible to remove these metal objects during examinations [ 24 , 29 ]. The process of debonding and rebonding of orthodontic brackets during treatment is not only costly and time-consuming, but it can also result in enamel damage and prolonging the overall treatment time [ 30 - 31 ]. Therefore, when an MRI examination is imperative, careful consideration of both the risks and benefits is essential to ensure that the optimal choice obtained for the patients [ 32 ]. While the effects of MRI on image quality and mechanical interactions with brackets have been well-documented, limited research has focused on its impact on microleakage [ 25 , 33 ].

The magnetic induction of the static magnetic field, which ranges from 0.2 to 9.4 tesla (T), plays a critical role in determining the clinical impact of MRI [ 34 ]. Thus, the extent of microleakage between the bracket and enamel interface is influenced by the magnetic field strength of MRI devices. With over 20,000 1.5T MRI systems currently in use, this field strength remains the most compatible for imaging the head and neck region [ 35 ]. Therefore, it is essential to investigate the microleakage beneath stainless steel brackets in comparison to titanium brackets, specifically within the 1.5T magnetic field.

Moreover, temperature changes induced by MRI exposure in orthodontic brackets have not been extensively studied. Although prior studies have shown minimal thermal effects under certain conditions, the impact of temperature changes on the adhesive interface remains unclear [ 25 , 36 ]. Understanding these interactions is crucial to ensuring that MRI examinations do not adversely affect orthodontic treatment.

Given the widespread use of 1.5 T MRI systems for head and neck imaging, this study aims to investigate and compare the effects of 1.5 T MRI on microleakage and temperature changes in stainless steel and titanium brackets, the most commonly used materials in fixed orthodontic treatments. The findings of this research will provide valuable insights into the safety and compatibility of these materials, offering guidance to clinicians who manage orthodontic patients undergoing MRI. The null hypothesis states that no significant differences exist in microleakage and temperature scores between titanium and stainless steel brackets following MRI scanning. 

## Materials and Method

This *in vitro* study was approved by Shiraz University of Medical Sciences (SUMS) Medical Ethics Committee (Approval ID:IR.SUMS.REC.1397.18). The sample size was determined using G*Power 3.1.9.2 software (Faul, Erdfelder, Buchner, &amp; Lang, 2014). Based on the study by Arikan *et al*. [ 37 ] a minimum of 40 teeth was required to achieve 90% statistical power with a significance level of 5% (α = 0.05) and an effect size of 1.10. Accordingly, 40 non-carious, recently extracted human maxillary premolars were selected. The soft tissue remnants and debris were removed, and the specimens were polished using non-fluoridated pumice paste and rubber cups for 10 seconds each. The teeth were then stored in distilled water at room temperature for one month. Prior to the bonding procedure, all specimens were disinfected by immersion in 1% thymol solution for one week. Teeth with fractures, enamel hypocalcifications, or abnormal surface morphology were excluded.

Two types of orthodontic brackets were used including stainless steel brackets (Victory Series™ Low Profile Bracket System, 3M Unitek, USA), and titanium brackets (Discovery Brackets, Dentaurum GmbH &amp; Co. KG, Germany).

Rectangular polyvinyl chloride (PVC) boxes, measuring 2cm in width, 8 cm in length, and 2 cm in depth, were prepared for each group. The teeth in each group were initially fixed side by side at the bottom of the PVC boxes, oriented perpendicular to the horizontal plane. Freshly mixed auto-polymerizing acrylic resin was then poured into the PVC boxes, filling them up to 2mm below the cemento-enamel junction. After polymerization, the acrylic blocks were carefully removed from the PVC boxes, and the buccal surfaces of the teeth were etched with 37% orthophosphoric acid gel (GC Ortho Etching Gel, GC, Japan) for 30 seconds. The surfaces were subsequently rinsed and dried with oil-free air. The bonding agent (Transbond XT, 3M Unitek, USA) was then applied, and light curing was performed for 20 seconds using a conventional LED device (Litex 696, Dentamerica, USA). The orthodontic brackets were located on the tooth surface using orthodontic adhesive (Transbond XT, 3M Unitek, USA) followed by light curing for 40 seconds (10 seconds on each side). This standardized protocol ensured uniform placement and consistent adhesive thickness across all samples.

**Figure 1:**
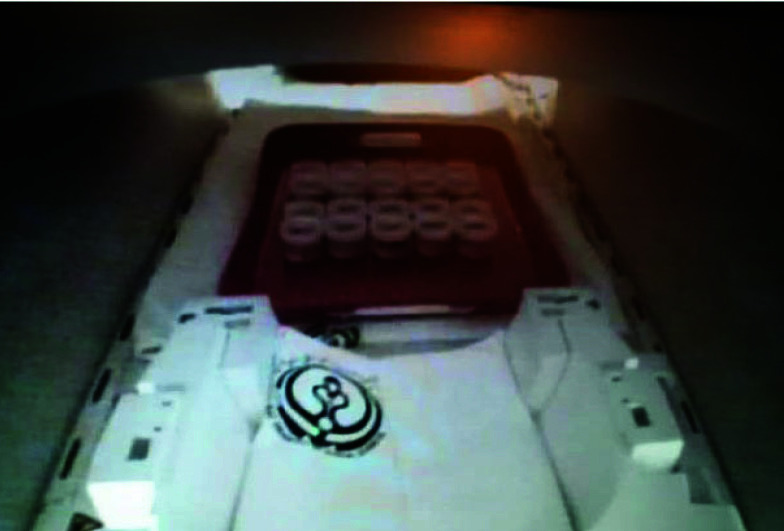
The case subgroups were exposed to 1.5 tesla MRI

The teeth were randomly divided into two groups (n= 20) based on bracket type, with each group further subdivided into control and case subgroups (n=10). The case subgroups were exposed to 1.5 T MRI using a 1.5 Tesla MRI unit (Avanto, Siemens, Germany) for 20 minutes, while the control subgroups were not exposed [ 38 ]. The MRI scanning parameters are outlined in Table 1. The samples in the case groups were positioned to maintain consistent bracket orientation during exposure (Figure 1). 

**Table 1:** MRI imaging parameters for the sequences performed at 1.5 Tesla

Imaging Sequence	TR (ms)	NEX	No. of slice	TE (ms)
T2	4170	2	30	91
T1	412	1	30	10
Flair	7500	1	30	102
T1 Sagittal	430	2	30	10
DWI axial	4500	4	30	100

An MRI-compatible fluoroptic four-channel thermometry system (Stanford Research SR630, Stanford, CA) was used to measure temperature changes associated with MRI exposure. Initially, the case subgroups were equilibrated at room temperature, and their base line temperatures were recorded. As soon as the 20-minute MRI scanning session concluded and the samples were safely removed from the MRI unit, the final temperature was recorded without any delay. Similarly, the control subgroups were assessed under comparable conditions at room temperature. Data acquisition for the thermometry probes was performed with a laptop computer and the dedicated software of the fluoroptic thermometry system. The thermometry probe was strategically positioned adjacent to the orthodontic appliance, ensuring accurate temperature readings with a precision of 0.1°C. 

**Figure 2:**
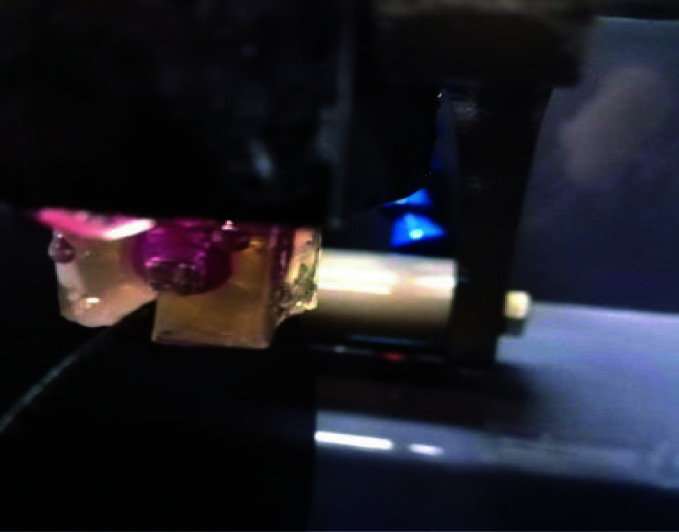
Sectioning of epoxy resin blocks was carried out using a low-speed diamond saw

**Figure 3:**
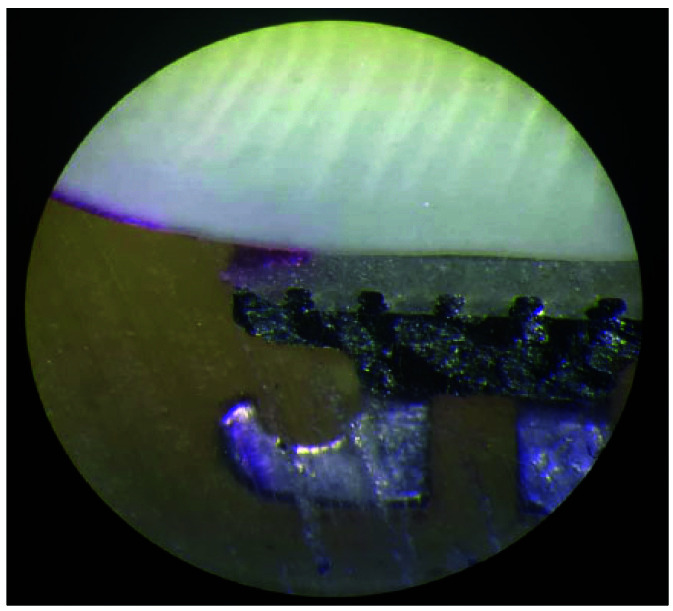
Samples were examined under a stereomicroscope at 40× magnification

The teeth were coated with two layers of nail varnish, leaving a 1mm margin around the bracket edges. Samples were immersed in 0.5% basic fuchsine dye solution (Labtron, Tehran, Iran) for 24 hours at room temperature. After removal from the solution, the teeth were rinsed with distilled water, the superficial dye was removed with a brush and the teeth were left to dry. The samples were embedded in epoxy resin blocks according to the direction of sections [ 39 ]. Sectioning was performed using a low-speed diamond saw (Figure 2). The sections were examined under a stereomicroscope at 40× magnification (Figure 3). The microleakage measurements were directly recorded using an electronic digital caliper (GuangLu Measuring Instrument Co. Ltd, Shanghai, China) by a single blinded observer. 

### Statistical analysis

Statistical analysis was conducted using SPSS software, version 15 (SPSS Inc., Chicago, IL). The normality of data was assessed using the Shapiro-Wilk test. Since the microleakage data were not normally distributed, the non-parametric Kruskal-Wallis test was used. In contrast, the temperature data followed a normal distribution and were analyzed using paired t-tests. Statistical significance was set at *p*< 0.05. 

## Results

A total of 40 extracted teeth were evaluated to measure temperature changes and assess microleakage at the bracket-adhesive and enamel-adhesive interfaces. The results are summarized in Tables 2 and 3. 

**Table 2:** The mean±SD of microleakage scores (mm) for occlusal/gingival bracket-adhesive and enamel-adhesive interfaces

Interface	Side	Experimental groups				*p* value
		3M (n:20)		Dentarum (n:20)		
		Control	Case	Control	Case	
Bracket/ Adhesive	Occlusal	0.10±0.02	0.11±0.02	0.11±0.03	0.10±0.02	0.545
	Gingival	0.12±0.09	0.11±0.03	0.12±0.02	0.11±0.03	0.513
Enamel/ Adhesive	Occlusal	0.27±0.06	0.31±0.07	0.26±0.06	0.30±0.08	0.386
	Gingival	0.23±0.08	0.27±0.07	0.28±0.07	0.29±0.08	0.452

**Table 3:** Comparison of temperature values (◦C) of different brackets before and after MRI exposure

Experimental groups	Before	After	Temperature changes	*p* Value
3M (n:10)	21.94±0.07	21.93±0.05	-0.006±0.045	0.685
Dentarum (n:10)	21.98±0.07	21.96±0.06	-0.013±0.048	0.149

### Microleakage Analysis

The microleakage scores for the occlusal and gingival sides of the bracket-adhesive and enamel-adhesive interfaces are presented in Table 2. Microleakage scores at the enamel-adhesive interface were slightly higher than at the bracket-adhesive interface, but the differences were not statistically significant (*p*> 0.05). No significant differences were observed between the stainless steel and titanium brackets in either the case (MRI-exposed) or control (non-exposed) groups (*p*> 0.05). These findings indicate that exposure to a 1.5 T MRI magnetic field did not significantly impact the adhesive integrity of either bracket type.

### Temperature Evaluation

Table 3 outlines the temperature changes measured before and after MRI exposure. Stainless steel brackets showed a minimal mean temperature change of -0.006± 0.045°C (*p*= 0.685). Titanium brackets exhibited a mean temperature change of -0.013±0.048°C (*p*= 0.149). No significant temperature changes were detected in either bracket type following MRI exposure (*p*> 0.05). These results suggest that 1.5 T MRI exposure does not induce clinically relevant thermal effects in orthodontic brackets. 

## Discussion

The interaction between fixed orthodontic appliances and MRI has been a topic of considerable interest, particularly concerning patient safety and the integrity of orthodontic treatment. This study aimed to evaluate the effects of 1.5 T MRI exposure on microleakage and temperature changes in stainless steel and titanium brackets. The findings suggested that exposure to 1.5 T MRI did not induce significant microleakage or temperature changes, supporting the notion of compatibility of these brackets with MRI.

Regardless of 1.5 Tesla MRI exposure, both titanium and stainless steel brackets exhibited similar results in terms of microleakage and thermal stability. These findings are consistent with those of Sfondrini *et al*. [ 38 ] who reported no effect on adhesion and stability following MRI exposure. Unlike their method of removing brackets for evaluation, this study assessed microleakage with brackets left in place. However, Bolat Gümüş *et al*. [ 40 ] noted significantly higher microleakage at 3 T MRI, indicating possible adhesion compromise at higher field strengths. Microleakage presents the likelihood of formation of white spot lesions so higher microleakage scores in 3 T MRI, may lead to enamel decalcification, white spot lesions and dental caries formation. Additionally, the increased microleakage can weaken the bracket–tooth bond, which may result in debonding during treatment. This not only delays the treatment process but also necessitates additional visits for rebonding, ultimately prolonging overall treatment time and increasing patient discomfort [ 41 ]. The higher the microleakage score, the greater the concerns about white spot lesions are. While this study provides reassurance regarding the safety of MRI exposure, it highlights the importance of monitoring orthodontic patients undergoing repeated MRI scans to ensure long-term adhesive integrity.

The slightly higher microleakage scores at the enamel-adhesive interface compared to the bracket-adhesive interface can be attributed to the differences in the thermal expansion coefficients of enamel and adhesive materials. However, the differences were not statistically significant, underscoring the stability of adhesive bonds under MRI exposure at 1.5 T.

Numerous techniques have been developed to investigate microleakage around dental restorations.

The most commonly used method involves exposing the specimens to a dye solution, followed by examination of the cross-sections using a light microscope [ 42 ]. The significance of a leakage test should be evaluated by considering the size of oral microorganisms. In this context, dyes such as fuchsine and methylene blue serve as excellent indicators for detecting clinically noticeable gaps [ 43 ]. Dye penetration was employed in this study due to its simplicity, cost-effectiveness, and ability to provide a quantitative measure of the extent of microleakage [ 44 ].

The selection of the fluoroptic thermometry system in our study was based on its precision (temperature resolution of 0.1°C) and the physical characteristics of its probe, which is both small and flexible- making it ideal for measuring localized temperature changes near orthodontic brackets. This feature was particularly important given the need for accurate assessment of thermal effects in a confined area during MRI exposure. Furthermore, the MRI compatibility and reliability of fluoroptic systems have been demonstrated in previous studies [ 36 , 45 ].

Temperature measurements revealed no significant changes before and after MRI exposure for either stainless steel or titanium brackets. While no statistically significant differences were observed, the consistent findings across bracket types increase the reliability of the study’s conclusions. This is consistent with findings from Linetskiy *et al*. [ 25 ] and Regier *et al*. [ 36 ], who reported negligible thermal effects under similar conditions. The minimal temperature fluctuations observed in this study fall well below the critical threshold for pulp vitality (5.6°C) [ 46 ]; indicating that MRI-induced heating is unlikely to affect dental tissues. Notably, under the specific scanning conditions of our study- including duration and sequence parameters- the observed temperature decrease may be attributed more to the environmental factors, such as ambient room temperature and the internal cooling systems of the MRI unit and MRI exposure alone was not sufficient to induce thermal change. A similar observation was reported by Regier *et al*. [ 36 ], who noted a slight temperature reduction during certain intervals of MRI scanning.

It is worth noting that some studies at higher MRI field strengths (e.g., 3 T) have reported more pronounced temperature increases, particularly in stainless steel brackets [ 15 , 47 ]. These differences could be attributed to variations in magnetic field strength, exposure duration, and appliance design. Although the temperature increase resulting from MRI exposure may not be sufficient to cause pulpal damage, it could potentially harm the oral mucosa and surrounding tissues. Therefore, it may be necessary to place a spacer between the appliance and the oral mucosa, or alternatively, remove the wire from the bracket prior to the MRI to minimize the risk of tissue damage [ 47 ].

One of the primary concerns of radiologists is the imaging artifact which obviously reduces the diagnostic quality of an image. Stainless steel can particularly be concerned, considering the ferromagnetic properties of iron and nickel [ 29 ]. Stainless steel wires used in orthodontics are safe in 1.5 T MRI but show significant deflection and torque at 3 T, affecting image quality. These wires can be removed before scanning [ 15 , 23 , 29 ]. However, fixed orthodontic brackets pose a greater challenge as they cannot be easily removed. Studies show stainless steel brackets severely distort MRI images, while ceramic, plastic, and titanium brackets cause minimal or no artifacts [ 23 - 24 , 48 ]. Artifact size decreases with greater distance between orthodontic appliances and the MRI target area [ 24 , 49 ]. Therefore, considering artifact production, titanium brackets are suitable for head and neck imaging, but stainless-steel brackets should be removed for scans like paranasal sinus MRI [ 49 ].

This study complements existing research by directly comparing stainless steel and titanium brackets under identical MRI conditions. Previous studies often focused on one material type or used varying methodologies, making comparisons difficult. Our findings confirm that titanium brackets, due to their non-ferromagnetic properties, exhibit comparable performance to stainless steel brackets at 1.5 T in terms of microleakage and thermal stability. These results suggest that both materials are safe for use in orthodontic patients undergoing head and neck MRI.

The findings have important clinical implications. The absence of significant microleakage or thermal changes suggests that fixed orthodontic appliances do not need to be removed prior to 1.5 T MRI examinations, provided image artifacts are not a concern. This reduces the need for debonding and rebonding of brackets, which can be time-consuming, costly, and potentially damaging to enamel. The primary stability of orthodontic brackets may be compromised in individuals undergoing repeated head-neck MRIs or in cases where prolonged use leads to a weakening of the composite bond, especially in patients nearing the end stages of orthodontic treatment. Therefore, it would be advisable to evaluate the stability of the brackets both before and after MRI exams in these patients. The decision to proceed with MRI should be individualized, considering potential image distortions caused by metallic brackets.

This study has some limitations. The controlled laboratory setting does not fully replicate clinical conditions, such as variations in oral temperature, saliva, and masticatory forces. Only one brand of stainless steel and titanium brackets were evaluated, which limits the generalizability of the findings to other products. Temperature changes were recorded only before and after MRI exposure. Continuous temperature monitoring during MRI exposure is recommended for future studies to capture transient thermal spikes.

Future research should address these limitations by conducting in vivo studies, evaluating a broader range of bracket brands and materials, and incorporating continuous temperature monitoring. Despite these limitations, the present study offers clinically relevant insights into the assessment of bond quality degradation in orthodontic brackets exposed to MRI. 

## Conclusion

The results of this study suggest that exposure to 1.5 T MRI does not significantly affect microleakage or temperature changes in stainless steel and titanium brackets. Provided that artifact formation is not a concern, continuing fixed orthodontic treatment after MRI imaging does not seem to increase the risk of white spot lesions caused by microleakage or pulp trauma resulting from temperature elevation. However, ongoing research is necessary to further elucidate the interactions between orthodontic materials and MRI under varied conditions.

## References

[1] Hubálková H, La Serna P, Linetskiy I, Dostálová TJ ( 2006). Dental alloys and magnetic resonance imaging. Int Dent J.

[2] Matthews PM, Jezzard P ( 2004). Functional magnetic resonance imaging. J Neurol Neurosurg Psychiatry.

[3] Shahidi S, Bronoosh P, Alavi A, Zamiri B, Sadeghi A, Bagheri M, et al ( 2009). Effect of magnetic resonance imaging on microleakage of amalgam restorations: an in vitro study. Dentomaxillofac Radio.

[4] Di Nardo D, Gambarini G, Capuani S, Testarelli L ( 2018). Nuclear magnetic resonance imaging in endodontics: a review. J Endo.

[5] Mendes S, Rinne CA, Schmidt JC, Dagassan-Berndt D, Walter C ( 2020). Evaluation of magnetic resonance imaging for diagnostic purposes in operative dentistry- a systematic review. Clin Oral Invest.

[6] Assaf AT, Zrnc TA, Remus CC, Schönfeld M, Habermann CR, Riecke B, et al ( 2014). Evaluation of four different optimized magnetic-resonance-imaging sequences for visualization of dental and maxillo-mandibular structures at 3 T. J Craniomaxillofa Surg.

[7] Ruf S, Pancherz H ( 1999). Temporomandibular joint remodeling in adolescents and young adults during Herbst treatment: a prospective longitudinal magnetic resonance imaging and cephalometric radiographic investigation. Am J Ortho Dentofac Orthop.

[8] Sabbagh H, Nikolova T, Kakoschke SC, Wichelhaus A, Kakoschke TK ( 2022). Functional Orthodontic Treatment of Mandibular Condyle Fractures in Children and Adolescent Patients: An MRI Follow-Up. Life (Basel).

[9] Sedlacik J, Kutzner D, Khokale A, Schulze D, Fiehler J, Celik T, et al ( 2016). Optimized 14+1 receive coil array and position system for 3D high-resolution MRI of dental and maxillomandibular structures. Dentomaxillofac Radio.

[10] Juerchott A, Freudlsperger C, Zingler S, Saleem MA, Jende JM, Lux CJ, et al ( 2020). In vivo reliability of 3D cephalometric landmark determination on magnetic resonance imaging: a feasibility study. Clin Oral Invest.

[11] Maspero C, Abate A, Bellincioni F, Cavagnetto D, Lanteri V, Costa A, Farronato M ( 2019). Comparison of a tridimensional cephalometric analysis performed on 3T-MRI compared with CBCT: a pilot study in adults. Prog Orthod.

[12] Kemper J, Priest AN, Schulze D, Kahl-Nieke B, Adam G, Klocke A ( 2007). Orthodontic springs and auxiliary appliances: assessment of magnetic field interactions associated with 1.5 T and 3 T magnetic resonance systems. Eur Radiol.

[13] Kajan ZD, Khademi J, Alizadeh A, Hemmaty YB, Roushan ZA ( 2015). A comparative study of metal artifacts from common metal orthodontic brackets in magnetic resonance imaging. Imag Sci Dent.

[14] Okano Y, Yamashiro M, Kaneda T, Kasai K ( 2003). Magnetic resonance imaging diagnosis of the temporomandibular joint in patients with orthodontic appliances. Oral Surg Oral Med Oral Patho Oral Radio Endo.

[15] Görgülü S, Ayyıldız S, Kamburoğlu K, Gökçe S, Ozen T ( 2014). Effect of orthodontic brackets and different wires on radiofrequency heating and magnetic field interactions during 3-T MRI. Dentomaxillofac Radio.

[16] Shellock FG, Spinazzi A ( 2008). MRI safety update 2008: part 2, screening patients for MRI. AJR Am J Roentgenol.

[17] Starčuková J, Starčuk Jr Z, Hubálková H, Linetskiy I ( 2008). Magnetic susceptibility and electrical conductivity of metallic dental materials and their impact on MR imaging artifacts. Dent Mate.

[18] Chockattu SJ, Suryakant DB, Thakur S ( 2018). Unwanted effects due to interactions between dental materials and magnetic resonance imaging: a review of the literature. Restor Dent Endod.

[19] Shalish M, Dykstein N, Friedlander-Barenboim S, Ben-David E, Gomori JM, Chaushu S ( 2015). Influence of common fixed retainers on the diagnostic quality of cranial magnetic resonance images. Am J Ortho Dentofac Orthop.

[20] Shellock FG ( 2002). Magnetic resonance safety update 2002: implants and devices. J Magn Reson Imaging.

[21] Gunzinger JM, Delso G, Boss A, Porto M, Davison H, von Schulthess GK, et al ( 2014). Metal artifact reduction in patients with dental implants using multispectral three-dimensional data acquisition for hybrid PET/MRI. EJNMMI Physics.

[22] Mundhada VV, Jadhav VV, Reche A ( 2023). A Review on Orthodontic Brackets and Their Application in Clinical Orthodontics. Cureus.

[23] Beau A, Bossard D, Gebeile-Chauty S ( 2017). Magnetic resonance imaging artefacts and fixed orthodontic attachments. L'Orthodontie Francaise.

[24] Elison JM, Leggitt VL, Thomson M, Oyoyo U, Wycliffe ND ( 2008). Influence of common orthodontic appliances on the diagnostic quality of cranial magnetic resonance images. Am J Ortho Dentofac Orthop.

[25] Linetskiy I, Starčuková J, Hubálková H, Starčuk Jr Z, Özcan M ( 2019). Evaluation of magnetic resonance imaging issues of titanium and stainless steel brackets. Science Asia.

[26] Zachriat C, Asbach P, Blankenstein K, Peroz I, Blankenstein F ( Dentomaxillofac Radio 2015). MRI with intraoral orthodontic appliance-a comparative in vitro and in vivo study of image artefacts at 1. 5 T.

[27] Yassi K, Ziane F, Bardinet E, Moinard M, Veyret B, Chateil JF ( 2007). Evaluation des risques d'échauffement et de déplacement des appareils orthodontiques en imagerie par résonance magnétique (Evaluation of the risk of overheating and displacement of orthodontic devices in magnetic resonance imaging). J Radiol.

[28] Alkis H, Turkkahraman H, Adanir N ( 2015). Microleakage under orthodontic brackets bonded with different adhesive systems. Eur J Dent.

[29] Hasanin M, Kaplan SE, Hohlen B, Lai C, Nagshabandi R, Zhu X, et al ( 2019). Effects of orthodontic appliances on the diagnostic capability of magnetic resonance imaging in the head and neck region: A systematic review. Int Ortho.

[30] Yassaei S, Aghili H, KhanPayeh E, Goldani Moghadam M ( 2014). Comparison of shear bond strength of rebonded brackets with four methods of adhesive removal. Lasers Med Sci.

[31] Degrazia FW, Genari B, Ferrazzo VA, Santos-Pinto AD, Grehs RA ( 2018). Enamel roughness changes after removal of orthodontic adhesive. Dent J.

[32] Zhylich D, Krishnan P, Muthusami P, Rayner T, Shroff M, Doria A, et al ( 2017). Effects of orthodontic appliances on the diagnostic quality of magnetic resonance images of the head. Am J Orthod Dentofacial Orthop.

[33] Kustarci A, Sokucu O ( 2010). Effect of chlorhexidine gluconate, Clearfil Protect Bond, and KTP laser on microleakage under metal orthodontic brackets with thermocycling. Photomed Laser Surg.

[34] Sasaki M, Inoue T, Tohyama K, Oikawa H, Ehara S, Ogawa A ( 2003). High-field MRI of the central nervous system: current approaches to clinical and microscopic imaging. Magn Reson Med Sci.

[35] DeLano MC, DeMarco JK (5 T MR Angiography of the head and neck Neuroimaging Clin N Am 2006). 3. 0 T versus 1.

[36] Regier M, Kemper J, Kaul MG, Feddersen M, Adam G, Kahl-Nieke B, et al ( J Orofac Orthop 2009). Radiofrequency-induced heating near fixed orthodontic appliances in high field MRI systems at 3. 0 Tesla.

[37] Arıkan S, Arhun N, Arman A, Cehreli SB ( 2006). Microleakage beneath ceramic and metal brackets photopolymerized with LED or conventional light curing units. Angle Ortho.

[38] Sfondrini MF, Preda L, Calliada F, Carbone L, Lungarotti L, Bernardinelli L, et al (2019). Magnetic resonance imaging and its effects on metallic brackets and wires: does it alter the temperature and bonding efficacy of orthodontic devices?. Materials.

[39] Pakshir H, Ajami S ( 2015). Effect of enamel preparation and light curing methods on microleakage under orthodontic brackets. J Dent (Tehran, Iran).

[40] Bolat Gümüş E, Şatir S, Kuştarci A ( 2022). Microleakage beneath orthodontic brackets in high field magnetic resonance imaging (MRI) AT 1.5 & 3 Tesla. Dentomaxillofac Radio.

[41] Arhun N, Arman A, Cehreli SB, Arıkan S, Karabulut E, Gülşahı K ( 2006). Microleakage beneath ceramic and metal brackets bonded with a conventional and an antibacterial adhesive system. Angle Orthod.

[42] Nilgun Ozturk  A, Usumez A, Ozturk B, Usumez S ( 2004). Influence of different light sources on microleakage of class V composite resin restorations. J Oral Rehabil.

[43] Hanks CT, Wataha JC, Parsell RR, Strawn SE, Fat JC ( 1994). Permeability of biological and synthetic molecules through dentine. J Oral Rehabil.

[44] Yap A, Stokes AN, Pearson GJ ( 1996). An in vitro microleakage study of a new multi-purpose dental adhesive system. J Oral Rehabil.

[45] Wezel J, Kooij BJ, Webb AG ( 2014). Assessing the MR compatibility of dental retainer wires at 7 Tesla. Magn Reson Med.

[46] Dobai A, Dembrovszky F, Vízkelety T, Barsi P, Juhász F, Dobó-Nagy C ( 2022). MRI compatibility of orthodontic brackets and wires: systematic review article. BMC Oral Health.

[47] Hasegawa M, Miyata K, Abe Y, Ishigami T ( 2013). Radiofrequency heating of metallic dental devices during 3.0 T MRI. Dentomaxillofac Radio.

[48] Zhylich D, Krishnan P, Muthusami P, Rayner T, Shroff M, Doria A, et al ( 2017). Effects of orthodontic appliances on the diagnostic quality of magnetic resonance images of the head. Am J Orthod Dentofac Orthop.

[49] Poorsattar-Bejeh Mir A, Rahmati-Kamel M (2016). Should the orthodontic brackets always be removed prior to magnetic resonance imaging (MRI)?. J Oral Biol Craniofac Res.

